# Supplementary material for: A manikin or human simulator—development of a tool for measuring students’ perception
Source: PeerJ. 2022 Dec 12;10:e14214. doi: 10.7717/peerj.14214 (PMC9753758; doi:10.7717/peerj.14214)
Supplement: Supplemental Information 1 [file peerj-10-14214-s001.docx]

Appendix 1 Semi-structured interview

***Dear S­­tudent,***

Medical Simulation (MS) is a technology supporting the learning process consisting in mirroring in the most realistic way the real clinical conditions. Medical Simulation is a method that has been utilized widely in health professions education. MS is standardized repeatable, and safe method of learning. One of the possible elements of this environment is the participation of Standardized Patients (patient actors mimicking diseases) in front of the learners (health profession students).

The aim of this interview is to find out/describe your opinion about learning with a manikin and standardized patient in an environment of high-fidelity scenarios. Your answers will help us to prepare a questionnaire regarding the evaluation and opinion of 6year Medical Students of the Medical University of Lublin about learning in high fidelity scenarios with manikin or standardized patients. A survey will be prepared based on your answers. The survey will help us in a better selection and participation of standardized patients or manikins.

The interview is anonymous and voluntary. Data obtained from this interview will be utilized only for scientific purposes.

I confirm I was informed about the purpose of this study. My comments will be anonymized and used only for the purpose of this research.

I consent to participate in the interview, and understand that I am free to withdraw at any time, without penalty.

Signed ......

Questions:

Participant code:

Date…………………………………………………

Year of studies……………………………….

Gender……………………………………………..

Age………………………………………………….

Clinical experience (other than in this course, such as nurse qualification)…………..

Have you participated in scenarios with standardized patients?

Have you participated in scenarios with manikins?

What are the advantages of learning with a manikin?

What are the disadvantages (limitations) of learning with a manikin?

What are the advantages of learning with a standardized patient?

What are the disadvantages of learning with a standardized patient?

What did you feel while examining a manikin compared to examining a standardized patient?

What was more helpful to learn from a scenario using a manikin or standardized patient?

What are the learning situations (procedures) that you find it more useful to be practiced with a manikin?

What are the learning situations (procedures) that you it more useful to be practiced with a standardized patient?

Have you been more careful while examining the manikin compared to examining a standardized patient?

How realistic was the simulation experience with a manikin?

How realistic was the simulation experience with a standardized patient?

Can you suggest a better alternative than the above mentioned simulation modalities?

Would you choose a different way of learning, if so what might that be?
