# Supplementary material for: A manikin or human simulator—development of a tool for measuring students’ perception
Source: PeerJ. 2022 Dec 12;10:e14214. doi: 10.7717/peerj.14214 (PMC9753758; doi:10.7717/peerj.14214)
Supplement: Supplemental Information 2 [file peerj-10-14214-s002.docx]

Appendix 2 Semi-structured interview (original version – Polish language)

***Szanowna Studentko, Szanowny Studencie,***

Symulacja medyczna jest technologią wspierającą uczenie polegającą na odzwierciedleniu w możliwie jak najwierniejszy sposób warunków rzeczywistych. Symulacja medyczna jest metodą coraz częściej znajdującą zastosowanie w kształceniu kadr medycznych. Jest to metoda wystandaryzowana, powtarzalna i bezpieczna dla uczącego się. Pewnym elementem symulacji medycznej jest udział pacjentów symulowanych (pacjentów-aktorów odgrywających sztuczne schorzenia) przed uczącymi się (studentami zawodów medycznych).

Celem tego wywiadu jest próba określenia Państwa doświadczeń uczenia się z pacjentem symulowanym i manekinem. Państwa odpowiedzi pozwolą na opracowanie kwestionariusza oceny i opinii studentów 6 roku lekarskiego UM w Lublinie uczenia się w środowisku scenariuszy wysokiej wierności z udziałem manekinów i pacjentów symulowanych. Przygotowana na tej podstawie ankieta pozwoli nam na lepszy dobór udziału pacjentów symulowanych i/lub manekinów w zajęciach wykorzystujących symulację medyczną.

Wywiad ma charakter dobrowolny, anonimowy. Uzyskane wyniki podlegają wglądowi na każdym etapie, a wyniki będą używane tylko do celów badawczych.

Imię ……………………………………………………

Nazwisko……………………………………………

Data…………………………………………………..

Rok studiów………………………………………

Ja niżej podpisany/a zostałem/a poinformowany/a o istocie badania.

Otrzymane informacje są wystarczające i wyrażam/nie wyrażam zgody na udział w wywiadzie.

Płeć

Wiek

Czy brałeś udział w zajęciach z pacjentem symulowanym?

Czy brałeś udział w zajęciach z manekinem?

Jakie Twoim zdaniem są zalety uczenia się z manekinem?

Jakie Twoim zdaniem są ograniczenia uczenia się z manekinem?

Jakie są zalety uczenia się z pacjentem symulowanym?

Jakie są ograniczenia uczenia się z pacjentem symulowanym?

Co czułeś, kiedy badałeś manekina, a co czułeś jak badałeś pacjenta symulowanego?

Czy uczenie się z manekinem lub pacjentem symulowanym uważasz za bardziej użyteczne?

Których procedur medycznych Twoim zdaniem byłoby się lepiej uczyć przy użyciu manekina?

Których procedur medycznych Twoim zdaniem byłoby się lepiej uczyć przy udziale pacjenta symulowanego?

Czy byłeś bardziej uważny podczas badania pacjenta symulowanego?

Jak oceniasz poziom realizmu scenariusza z udziałem manekina?

Jak oceniasz poziom realizmu scenariusza z udziałem pacjenta symulowanego?

Czy znasz inne lepsze alternatywy dla manekina lub pacjenta symulowanego?

Czy znasz inne formy uczenia, jeśli tak to jakie?
