# Supplementary material for: A manikin or human simulator—development of a tool for measuring students’ perception
Source: PeerJ. 2022 Dec 12;10:e14214. doi: 10.7717/peerj.14214 (PMC9753758; doi:10.7717/peerj.14214)
Supplement: Supplemental Information 3 [file peerj-10-14214-s003.docx]

Appendix 3 Questionnaire survey – preliminary version of 33 statements

| Program of study/ Major | Year of study | Sex | Age |
| --- | --- | --- | --- |
|  |  |  |  |

Dear Students,

We kindly ask you to provide reliable answers to the survey questions, which will be used to evaluate the effectiveness of manikin-based high fidelity simulation scenarios for learning clinical objectives. The participation in the survey is voluntary and anonymous.

Please answer the following questions using YES/NO option.

Questions about manikin:

1. Using manikin-based simulation encourages development of communication skills more than SP-based simulation.

| - Yes | - No |
| --- | --- |

1. Using manikin-based simulation enables learning about various diagnostic and therapeutic procedures more than SP-based simulation.

| - Yes | - No |
| --- | --- |

1. Using manikin-based simulation enables learning rare and pathological cases more than SP-based simulation.

| - Yes | - No |
| --- | --- |

1. Using manikin-based simulation enables technical skills development more than SP-based simulation.

| - Yes | - No |
| --- | --- |

1. Using manikin-based simulation limits the sense of realism of particular clinical cases more than SP-based simulation.

| - Yes | - No |
| --- | --- |

1. Manikin-based simulation can help in the process of learning patient history taking more than SP-based simulation.

| - Yes | - No |
| --- | --- |

1. Manikin-based simulation enhances appropriate professional performance when performing medical procedures more than SP-based simulation.

| - Yes | - No |
| --- | --- |

1. Physical examination performed on a manikin enables better understanding of patient reactions than the one performed on an SP.

| - Yes | - No |
| --- | --- |

1. Manikin-based simulation enables better understanding of patient rights more than SP-based simulation.

| - Yes | - No |
| --- | --- |

1. Communication during manikin-based simulation classes helps to develop proper doctor – patient rapport more than during SP-based classes.

| - Yes | - No |
| --- | --- |

1. The scope of possible medical procedures is limited during manikin-based simulation classes more than during SP-based classes.

| - Yes | - No |
| --- | --- |

1. Manikin-based simulation enables development of non-technical skills more than SP-based simulation.

| - Yes | - No |
| --- | --- |

1. The lack of possibility to observe patient subjective reactions during physical examination limits the reliability of the case and, thereby, of the learning process, during manikin-based simulation.

| - Yes | - No |
| --- | --- |

1. Manikin-based simulated scenario is more realistic than SP-based scenario.

| - Yes | - No |
| --- | --- |

Questions about SP:

1. Using SP-based simulation helps eliminating mistakes when performing medical procedures more than manikin-based simulation.

| - Yes | - No |
| --- | --- |

1. Using SP-based simulation enhances team work and management more than manikin-based simulation.

| - Yes | - No |
| --- | --- |

1. Using SP-based simulation encourages development of communication skills more than manikin-based simulation.

| - Yes | - No |
| --- | --- |

1. Using SP-based simulation enables learning about various diagnostic and therapeutic procedures more than manikin-based simulation.

| - Yes | - No |
| --- | --- |

1. Using SP-based simulation enables learning rare and pathological cases more than manikin-based simulation.

| - Yes | - No |
| --- | --- |

1. Using SP-based simulation enables technical skills development more than manikin-based simulation.

| - Yes | - No |
| --- | --- |

1. Using SP-based simulation limits the sense of realism of particular clinical cases more than manikin-based simulation.

| - Yes | - No |
| --- | --- |

1. The responsiveness of an SP affects the fluency of interaction and making decisions.

| - Yes | - No |
| --- | --- |

1. SP-based simulation can help in the process of learning patient history taking more than manikin-based simulation.

| - Yes | - No |
| --- | --- |

1. SP-based simulation enhances appropriate professional performance when performing medical procedures more than manikin-based simulation.

| - Yes | - No |
| --- | --- |

1. Physical examination performed on an SP enables better understanding of patient reactions than the one performed on a manikin.

| - Yes | - No |
| --- | --- |

1. SP-based simulation enables better understanding of patient rights more than manikin-based simulation.

| - Yes | - No |
| --- | --- |

1. Communication during SP-based simulation classes helps to develop proper doctor – patient rapport more than during manikin-based classes.

| - Yes | - No |
| --- | --- |

1. The lack of possibility to present rare and pathological findings in an SP limits the range of diagnostic and treatment procedures that can be learnt.

| - Yes | - No |
| --- | --- |

1. SP-based simulation enables development of non-technical skills more than manikin-based simulation.

| - Yes | - No |
| --- | --- |

1. The possibility to feel the real body of a patient and observe his/her reactions to touch during physical examination increases the reliability of the case and, thereby, of the learning process, during SP-based simulation.

| - Yes | - No |
| --- | --- |

1. The possibility to observe patient subjective reactions during physical examination increases the reliability of the case and, thereby, of the learning process, during SP-based simulation?

| - Yes | - No |
| --- | --- |

1. SP-based simulated scenario is more realistic than manikin-based scenario.

| - Yes | - No |
| --- | --- |

1. The diversity of SP’s looks and behaviour can affect the sense of realism during simulation scenario?

| - Yes | - No |
| --- | --- |
