# Supplementary material for: A manikin or human simulator—development of a tool for measuring students’ perception
Source: PeerJ. 2022 Dec 12;10:e14214. doi: 10.7717/peerj.14214 (PMC9753758; doi:10.7717/peerj.14214)
Supplement: Supplemental Information 4 [file peerj-10-14214-s004.docx]

Appendix 4 Questionnaire survey – preliminary version of 33 statements (original version – Polish language)

| Kierunek studiów: | Rok studiów: | Płeć: K / | M | Wiek: |
| --- | --- | --- | --- | --- |

Szanowna Studentko, Szanowny Studencie,

Zwracamy się z uprzejmą prośbą o rzetelne wypełnienie ankiety, która zostanie użyta do oceny skuteczności nauki z wykorzystywaniem scenariuszy symulacji wysokiej wierności z udziałem pacjentów symulowanych [SP] oraz manekinów. Udział w ankiecie jest dobrowolny i anonimowy.

**Proszę o udzielenie odpowiedzi na poniższe pytania wybierając odpowiedź TAK lub NIE.**

Pytania dotyczące manekina:

1. Zajęcia symulacyjne z udziałem manekina bardziej niż z udziałem SP umożliwiają rozwój umiejętności komunikowania się z pacjentem.

| - TAK | - NIE |
| --- | --- |

1. Zajęcia symulacyjne z udziałem manekina bardziej niż z udziałem SP umożliwiają naukę różnych procedur diagnostycznych i terapeutycznych.

| - TAK | - NIE |
| --- | --- |

1. Zajęcia symulacyjne z udziałem manekina bardziej niż z udziałem SP dają większą możliwość poznawania zjawisk rzadkich i patologicznych.

| - TAK | - NIE |
| --- | --- |

1. Zajęcia symulacyjne z udziałem manekina bardziej niż z udziałem SP umożliwiają rozwój umiejętności technicznych.

| - TAK | - NIE |
| --- | --- |

1. Udział podczas zajęć manekina bardziej niż udział SP ogranicza poczucie realizmu danego przypadku klinicznego.

| - TAK | - NIE |
| --- | --- |

1. Udział w zajęciach manekina bardziej niż udział SP może pomóc w nauce zbierania wywiadu.

| - TAK | - NIE |
| --- | --- |

1. Udział w zajęciach manekina bardziej niż udział SP wspomaga bardziej profesjonalne zachowanie podczas wykonywanych procedur.

| - TAK | - NIE |
| --- | --- |

1. Badanie fizykalne wykonane na manekinie bardziej niż to wykonane na SP pozwala lepiej zrozumieć odczucia pacjenta.

| - TAK | - NIE |
| --- | --- |

1. Udział w zajęciach manekina bardziej niż udział SP pozwala lepiej zrozumieć prawa pacjenta.

| - TAK | - NIE |
| --- | --- |

1. Komunikacja podczas zajęć z manekinem bardziej niż komunikacja podczas zajęć z SP uczy jak budować relacje z pacjentem.

| - TAK | - NIE |
| --- | --- |

1. W trakcie zajęć symulacyjnych z udziałem manekina bardziej ograniczony jest zakres procedur medycznych możliwych do wykonania niż w trakcie zajęc z udziałem SP.

| - TAK | - NIE |
| --- | --- |

1. Udział w zajęciach manekina bardziej niż udział SP umożliwia rozwój umiejętności nietechnicznych.

| - TAK | - NIE |
| --- | --- |

1. Brak możliwości czucia ciała prawdziwego pacjenta oraz obserwacji jego reakcji na dotyk zmniejsza wiarygodność sytuacji i tym samym całego procesu uczenia się podczas zajęć z manekinem.

| - TAK | - NIE |
| --- | --- |

1. Scenariusz symulacyjny jest odbierany jako bardziej realistyczny jeśli realizowany jest z udziałem manekina a nie z udziałem SP.

| - TAK | - NIE |
| --- | --- |

Pytania dotyczące manekina:

1. Zajęcia symulacyjne z udziałem SP bardziej niż z udziałem manekina umożliwiają pracę nad eliminowaniem błędów podczas wykonywania procedur medycznych.

| - TAK | - NIE |
| --- | --- |

1. Zajęcia symulacyjne z udziałem SP bardziej niż z udziałem manekina umożliwiają naukę pracy w zespole.

| - TAK | - NIE |
| --- | --- |

1. Zajęcia symulacyjne z udziałem SP bardziej niż z udziałem manekina umożliwiają rozwój umiejętności komunikowania się z pacjentem.

| - TAK | - NIE |
| --- | --- |

1. Zajęcia symulacyjne z udziałem SP bardziej niż z udziałem manekina umożliwiają naukę różnych procedur diagnostycznych i terapeutycznych.

| - TAK | - NIE |
| --- | --- |

1. Zajęcia symulacyjne z udziałem SP bardziej niż z udziałem manekina dają większą możliwość poznawania zjawisk rzadkich i patologicznych.

| - TAK | - NIE |
| --- | --- |

1. Zajęcia symulacyjne z udziałem SP bardziej niż z udziałem manekina umożliwiają rozwój umiejętności technicznych.

| - TAK | - NIE |
| --- | --- |

1. Udział podczas zajęć SP bardziej niż udział manekina ogranicza poczucie realizmu danego przypadku klinicznego.

| - TAK | - NIE |
| --- | --- |

1. Responsywność SP może wpływać na płynność interakcji i podejmowanych decyzji.

| - TAK | - NIE |
| --- | --- |

1. Udział w zajęciach SP bardziej niż udział manekina może pomóc w nauce zbierania wywiadu.

| - TAK | - NIE |
| --- | --- |

1. Udział w zajęciach SP bardziej niż udział manekina wspomaga bardziej profesjonalne zachowanie podczas wykonywanych procedur.

| - TAK | - NIE |
| --- | --- |

1. Badanie fizykalne wykonane na SP bardziej niż to wykonane na menekinie pozwala lepiej zrozumieć odczucia pacjenta.

| - TAK | - NIE |
| --- | --- |

1. Udział w zajęciach SP bardziej niż udział manekina pozwala lepiej zrozumieć prawa pacjenta.

| - TAK | - NIE |
| --- | --- |

1. Komunikacja podczas zajęć z SP bardziej niż komunikacja podczas zajęć z manekinem uczy jak budować relacje z pacjentem.

| - TAK | - NIE |
| --- | --- |

1. Brak objawów rzadkich i patologicznych u SP ogranicza możliowść wykonywania określonych procedur diagnostycznych i terapeutycznych.

| - TAK | - NIE |
| --- | --- |

1. Udział w zajęciach SP bardziej niż udział manekina umożliwia rozwój umiejętności nietechnicznych.

| - TAK | - NIE |
| --- | --- |

1. Fakt możliwości czucia ciała prawdziwego człowieka oraz obserwacji jego reakcji na dotyk zwiększa wiarygodność sytuacji i tym samym całego procesu uczenia się podczas zajęć z SP.

| - TAK | - NIE |
| --- | --- |

1. Fakt możliwości obserwacji subiektywnych reakcji prawdziwego człowieka w trakcie badania zwiększa wiarygodność sytuacji i całego procesu uczenia się podczas zajęć z SP.

| - TAK | - NIE |
| --- | --- |

1. Scenariusz symulacyjny jest odbierany jako bardziej realistyczny jeśli realizowany jest z udziałem SP a nie z udziałem manekina.

| - TAK | - NIE |
| --- | --- |

1. Fakt różnorodności wyglądu i zachowań SP może mieć wpływ na poczucie realizmu podczas zajęć opartych na symulacji medycznej.

| - TAK | - NIE |
| --- | --- |
