# Supplementary material for: A manikin or human simulator—development of a tool for measuring students’ perception
Source: PeerJ. 2022 Dec 12;10:e14214. doi: 10.7717/peerj.14214 (PMC9753758; doi:10.7717/peerj.14214)
Supplement: Supplemental Information 5 [file peerj-10-14214-s005.docx]

| Demographics - Program of study (column B) | 1 - medicine |
| --- | --- |
| Demographics – Sex (column D) | 1 - woman; 2 - man |
| Questions 1-46 (column F-AY) | 1 – NO 2 – YES |
